# Surgical Implications of Implanted Cardiac Rhythm Devices

**DOI:** 10.19102/icrm.2023.14015

**Published:** 2023-01-15

**Authors:** Emily Collins, Miranda Marcucci, Jeffrey C. Liu, Andy C. Kiser

**Affiliations:** ^1^Perfusion Division, St. Clair Health, Pittsburgh, PA, USA; ^2^Lake Erie College of Osteopathic Medicine, Erie, PA, USA; ^3^St. Clair Health, Pittsburgh, PA, USA; ^4^Department of Cardiothoracic Surgery, University of Pittsburgh School of Medicine, Pittsburgh, PA, USA

**Keywords:** Cardiac implantable electronic device, consensus statement, electromagnetic interference, surgery

All surgeons, particularly cardiovascular surgeons, encounter patients with cardiac implantable electronic devices (CIEDs). In 2022, the European Heart Rhythm Association (EHRA), in collaboration with the Heart Rhythm Society (HRS), Latin America Heart Rhythm Society (LAHRS), and the Asia-Pacific Heart Rhythm Society (APHRS), published their consensus statement on the prevention and management of interference due to medical procedures in patients with CIEDs.^1^ The authors provide a comprehensive analysis of how external interferences impact the function of implanted electrical devices, such as pacemakers, implantable cardioverter defibrillators, cardiac resynchronization therapy devices and implantable loop recorders. We review this important article from 2022 with attention to vital considerations for surgeons performing procedures on patients with CIEDs.

## Summary of the consensus statement

The EHRA consensus was developed by a panel of 20 authors and representatives from HRS, LAHRS, and APHRS who performed a thorough review of published literature in order to develop a categorized and critically supported consensus document. The EHRA consensus was built upon the well-established practice that CIED patients demand distinct consideration throughout the perioperative period with a “no-one-size-fits-all” approach for the procedural considerations fundamental to each device type. Individual devices and manufacturers have unique properties and programming, which surgeons should understand when treating CIED patients. The EHRA consensus developed measured recommendations with associated color-coded categories. Statements supported by strong scientific evidence and expert agreement (green) should be universally followed. Those with general agreement and moderate supporting evidence (yellow) should be regularly implemented. Finally, procedures or testing that should not be used (red) based on published evidence and expert agreement should be avoided.

## Descriptions and actions of cardiac implantable electronic devices

### Pacemakers

The development of the pacemaker in the 1960s had a substantial impact on the interventions available to the cardiac patient. These devices sense aberrations within the electrical activity of the heart and, as a result, send out support signals on demand to induce myocardial contraction. Given the nature of how these devices work, the support they provide is inherently at risk of interference from outside electromagnetic sources, which they may incorrectly attribute as originating from the heart’s conduction system. This interaction has created clinical dilemmas for health care providers, especially with the increasing population of CIED patients.^2^ While modern pacemakers have been developed to withstand most normal day-to-day electromagnetic interference, the hospital setting is still associated with an increased risk to these devices and the patients they support. Electromagnetic interference (EMI) affecting these devices may result in pacing inhibition, cross-chamber pacing, alteration of rate responses, asynchronous pacing, inappropriate mode switching, modification of pacing thresholds, pacemaker-induced tachycardia and/or permanent effects on power on reset and backup mode.^1^

### Implantable cardioverter-defibrillators

Implantable cardioverter-defibrillators (ICDs) also sense aberrations within the heart’s electrical activity and will shock the heart in order to reset a rhythm that is too fast to support hemodynamic stability. Modern ICDs also have pacemaker functionality. Oversensing caused by EMI can result in inappropriate shocks to the patient, modified pacing function, long–short–long sequence pacing, truncation of pacing output, and/or sudden battery depletion.

### Cardiac resynchronization therapy devices

For patients with low ejection fractions (<35%) and prolonged QRS durations (>150 ms) with left bundle branch block, cardiac resynchronization therapy (CRT) may improve cardiac output and heart failure symptoms. CRT re-establishes the electrical coordination of the left and right ventricular chambers using sophisticated pacemaker algorithms. In addition to transvenous lead sensing and pacing of the right ventricle, a second ventricular lead senses and paces the left ventricle. The left ventricular lead most commonly resides within the distal branch veins of the coronary sinus, but it may also be located on the epicardial surface of the left ventricle. The implanted device may also include an ICD capability.

### Implantable loop recorders

Patients with implantable loop recorders that experience EMI may be subject to artifacts that mimic tachyarrhythmias.

## Sources of interference during surgical procedures

Electromagnetic fields (EMFs) and radiation are known to cause interference with CIEDs and may pose a significant risk to patients who are supported by these devices. Possible effects include direct damage to the leads or devices, oversensing resulting in either lack of therapeutic stimulation or inappropriate pacing/ICD therapy,^1^ tissue burns, induced atrial or ventricular fibrillation, or device reprogramming.^2^ Device interference may be caused by direct contact or by radiated interference. Common sources of interfering electromagnetic fields include electrocautery, radiofrequency ablation (both cardiac and non-cardiac), radiofrequency identification devices, electrical stimulation therapy, and therapeutic diathermy. Electrical cardioversion/defibrillation, electroconvulsive therapy, magnet-utilizing tissue expanders, electromyograms, and lithotripsy have also been documented as sources of interference. Magnetic resonance imaging (MRI) and exposure to therapeutic ionizing radiation (gamma rays, photon and proton beams, and carbon ions) are the most common radiation sources of interference with CIEDs. The hospital setting and surgical procedures expose patients to these frequencies, and it is vital that physicians and other health care clinicians are aware of the steps to mitigate significant negative outcomes.

## Management of patients with implanted cardiac devices during surgical procedures

Perhaps the most important aspect of the perioperative management of the patient with a CIED is collaboration with the cardiologist, specifically the electrophysiologist. Whenever possible, CIED patients should undergo a cardiology evaluation prior to any procedure, especially one with anticipated exposure to device interference. The pre-procedural evaluation should include a thorough work-up of the patient’s device type, model, and manufacturer. A chest X-ray may be required in order to determine details of the device, leads, and placement. A review of the device’s functionality, battery status, program settings, and the patient’s reliance on device interventions needs to be completed to properly plan and progress to the operative phase of the patient’s hospital course. Once the patient’s device status is well understood, the operative details may be assessed to determine the appropriate course of action for safe procedure performance. The approach will vary depending on the device, area of planned treatment, patient positioning, and anticipated exposure to EMI.

Intraoperatively, electrocardiographic monitoring is important, particularly if the use of electrocautery is anticipated. Participating clinicians should be attuned to possible EMI and ensure optimized monitor settings for CIED patients. The ability to monitor peripheral pulses is also a necessity for identifying any hemodynamic instability. Defibrillation and grounding pads should be placed >15 cm away from the device to avoid permanent damage in the event backup pacing or defibrillation is required. Defibrillation pads should be positioned in the location that directs the vector of delivered energy away from the CIED, generally in an anterior-to-posterior manner. At least 1 (but ideally 2) magnet(s) (≥10 G) should be on hand at all times during the procedure. In addition, personnel trained on the specific CIED device need to be available in-house should device reprogramming be required.

Electrocautery is the main source of EMI while in the operating room. Though intermittent electrocautery application is recommended, i.e., employing <5-s cautery bursts, surgeons should understand that this does not totally eliminate the risk of ICD shocks. For this reason, if available, the use of bipolar electrocautery and/or ultrasonic scalpels is advised. The EHRA consensus statement highlights that, while previous recommendations have indicated that ICD inactivation may be avoided in patients being treated below the umbilicus if unipolar electrocautery is utilized, unintentional misplacement (return pad too close to device) or utilization of full-body return electrode pads (Megadyne™; Johnson and Johnson, New Brunswick, NJ, USA) has resulted in device oversensing. Updated guidance suggests that the iliac crest is a more suitable demarcation, with the return electrode placed on the thigh, and the EHRA consensus statement thus references this rather than the umbilicus. Following this guidance, magnet application and/or device reprogramming, in general, is not required for procedures performed below the iliac crest. The consensus statement, however, does indicate that, while not mandatory, it may simplify the protocol to universally plan on magnet application for all ICD patients regardless of treatment location. Device reprogramming prior to any procedure with the slightest risk of EMI is necessary for all pacemaker-dependent ICD patients.

Optimally, and prior to the patient entering the procedure room, an experienced practitioner should reprogram the device or at least determine whether the procedure and/or device can safely proceed without reprogramming. Industry-employed professionals should not develop treatment strategies. Although reprogramming to the asynchronous mode may be the most common intervention, each device and clinical situation warrants an individualized assessment and modification. Magnet application for pacemakers does not ensure a standardized response and varies by specific device **(Table 1)**. While magnet application results in asynchronous pacing, ideal magnet placement and default magnet mode settings vary depending on manufacturer, with some having only a temporary asynchronous response. The ability for the device to spontaneously reprogram intraoperatively in response to EMI should not be overlooked. Continuous electrocardiogram monitoring is critical throughout the time period in which pacemaker patients have an applied magnet or asynchronous mode set, as there is a risk of ventricular pro-arrhythmia, which may require external defibrillation and transcutaneous pacing. Pacemaker-dependent ICD patients must have their device reprogrammed for the intraoperative period, as pacing mode will not be affected by magnet application. When evaluating cardiac surgical patients, procedures are typically done ≤15 cm from CIED devices, and devices should all be reprogrammed for the procedures’ entirety. This guidance is also applicable in the setting of procedures performed <15 cm away with secure magnet placement impossible due to patient positioning.

Immediately following any procedure, the CIED needs to be interrogated for any perioperative events and reprogramming. Procedures with EMI involvement must receive a full device follow-up by the electrophysiologist to ensure no physical damage or dysfunction has occurred. Patients should be closely monitored with appropriate interventions immediately available until proper review and reprogramming occurs.

To summarize, the safest recommendations include reprogramming the CIED prior to a surgical procedure and continuously monitoring electrocardiography and blood pressure. These management plans help reduce the risk of human error with pad or magnet placement, low awareness of EMI risk, inadequate assessment of pacing dependence, or unsuccessful memorization of multiple variables and flow charts. Clinical decisions regarding perioperative CIED management may be based upon the perception of risk or device dependency, which can only be truly assessed by device interrogation. These assumptions and recommendations also do not include any element of patient or procedure variability. One cannot always predict how a patient will react while under anesthesia or anticipate unexpected surgical events.

## Figures and Tables

**Table 1:** Effect of Magnet Placement on Cardiac Implantable Electronic Devices by Manufacturer^3^

Manufacturer	Device Response (No ICD)	ICD Response	ICD Audible Signal	Comments
Abbott	Pace at 100	Detection and Defibrillation off	No	When AutoCapture is enabled, remains on high output mode
Biotronik	Pace at 90 for 10 beats, then return to the programmed mode	Detection off	No	Magnet application may only last for 8 h, even with magnet still in place
Boston Scientific	Pace at 100	Defibrillation off	Yes	N/A
Medtronic	Pace at 85	Detection and Defibrillation off	Yes	No magnet response for Micra™ leadless pacemaker; magnet application may be ignored if placed within 1 h of interrogation
Microport/Sorin	Pace at 96	Detection off	No	With Platinium™ devices, magnet rate is not enabled

*Abbreviations:* ICD, implantable cardioverter-defibrillator.
